# Microsatellite Marker Analysis Reveals the Complex Phylogeographic History of *Rhododendron ferrugineum* (Ericaceae) in the Pyrenees

**DOI:** 10.1371/journal.pone.0092976

**Published:** 2014-03-25

**Authors:** Olivia Charrier, Pierre Dupont, André Pornon, Nathalie Escaravage

**Affiliations:** 1 University Toulouse III Paul Sabatier, Lab Evolution & Diversite Biologique EDB, F-31062 Toulouse, France; 2 CNRS, EDB, UMR 5174, F-31062 Toulouse, France; CNR, Italy

## Abstract

Genetic variation within plant species is determined by a number of factors such as reproductive mode, breeding system, life history traits and climatic events. In alpine regions, plants experience heterogenic abiotic conditions that influence the population's genetic structure. The aim of this study was to investigate the genetic structure and phylogeographic history of the subalpine shrub Rhododendron ferrugineum across the Pyrenees and the links between the populations in the Pyrenees, the Alps and Jura Mountains. We used 27 microsatellite markers to genotype 645 samples from 29 Pyrenean populations, three from the Alps and one from the Jura Mountains. These data were used to estimate population genetics statistics such as allelic richness, observed heterozygosity, expected heterozygosity, fixation index, inbreeding coefficient and number of migrants. Genetic diversity was found to be higher in the Alps than in the Pyrenees suggesting colonization waves from the Alps to the Pyrenees. Two separate genetic lineages were found in both the Alps and Pyrenees, with a substructure of five genetic clusters in the Pyrenees where a loss of genetic diversity was noted. The strong differentiation among clusters is maintained by low gene flow across populations. Moreover, some populations showed higher genetic diversity than others and presented rare alleles that may indicate the presence of alpine refugia. Two lineages of R. ferrugineum have colonized the Pyrenees from the Alps. Then, during glaciation events R. ferrugineum survived in the Pyrenees in different refugia such as lowland refugia at the eastern part of the chain and nunataks at high elevations leading to a clustered genetic pattern.

## Introduction

The maintenance of genetic diversity within populations is essential for the conservation of species since it can enable adaptation to future environmental changes [1], [2]. With high genetic diversity, it is more likely that some individuals will possess alleles that could allow the population to adapt to a new habitat [3]. High genetic diversity may be maintained via gene flow among individuals and among populations. In recent years, many studies have shown strong impacts of global change on species' genetic diversity [4]. Because climate warming is greater at high than at low elevations [5] alpine biodiversity hotspots that are home to many endemic and rare species may be particularly threatened by climate change [6]. Thus, to predict how these biodiversity hotspots will deal with climate changes, it is important to understand the genetic patterns of the species and their changes over time [7].

Genetic structure and genetic divergence within and among plant populations result from a number of contemporary and historical factors acting at various temporal and spatial scales. Alpine habitats are typically characterized by small spatial changes in environmental variables such as topography, temperature, snow cover, soil moisture or bedrock [8] favorable to phenotypic differentiation and local genetic variability over short geographical distances. In addition to abiotic factors, life history traits can also influence genetic features of populations. For instance, selfing perennials and clonal species generally exhibit lower levels of genetic diversity and higher levels of population differentiation than outcrossing perennials [9]. In contrast, mixed mating systems maintain a high level of genetic diversity and may be advantageous for plants in variable alpine environments [6]. The combined effects of both life history traits and small-scale abiotic heterogeneity may result in local genetic adaptations and high genetic differentiation of populations, notably where gene exchanges between populations are restricted as is often the case in mountain habitats [10]. Therefore, plant-community fragmentation pattern, steep climatic gradients leading to asynchronous flowering phenology or disjoint pollinator communities [7], and topographical barriers may hamper gene flow (through seed and pollen movements) and may increase differentiation between populations.

At large scales, species genetic patterns are often shaped by past climate-driven range dynamics [11]. In temperate regions, species ranges have changed dramatically over the Pleistocene with its successions of glaciations and warming periods. In mountains, periodic advances and retreats of glaciers have, on several occasions, forced species to descend to lowland and peripheral refugia and/or to survive in isolated ice-free areas above glaciers (nunataks). Genetic isolation, bottlenecks and potential allopatric evolution of populations in refugia have been followed by repeated founding events during population expansions, which have led to loss of genetic diversity and heterozygosity [11]. Additionally, populations located at eroding margins of species distribution ranges following climate warming could have persisted in suitable isolated lowland habitats [12]. It has been suggested [13]–[14] that such small populations isolated for so long could display low within-population genetic diversity (as a result of genetic drift) but have disproportionally high levels of between-population differentiation and regional genetic diversity and distinctiveness [15].

The genetic pattern and phylogeography of many species [16], [17] have been investigated in various mountainous regions, but to date, only a very limited number of studies have focused on the Pyrenees [18]–[20]. The Pyrenees are a mountain range stretching across the isthmus that lies between the Iberian Peninsula and the rest of the European continent. Acting as a biogeographical barrier during postglacial expansions, they are a limit for numerous Iberian and northern species. However, lower lands at the eastern and western extremities of the mountain range have been corridors for migration of several plant and animal species [21], [22]. Due to oceanic influences to the west and Mediterranean influences to the east of the chain, the climate varies greatly along a longitudinal gradient in the Pyrenees (Météo France data). This bioclimatic pattern potentially allowed some species to survive glaciations in various refugia along the chain. Studies have suggested that species have survived glaciations [23]–[25] in two main refugia - one in south central and another in the eastern Pyrenees - thanks to mild Mediterranean and oceanic climatic influences. However both geological and biological data [26] demonstrate that during glacial periods the ice sheet did not completely cover the highlands and rarely descended below 1000 m a.s.l except for big glaciers pushed down into large valleys and to the plains [23]. These nunataks and unglaciated peripheral area could have provided refugia for some species [18] and determined several recolonizing routes sculpturing a specific and complex genetic structure in the Pyrenees.

Here we investigate the genetic structure and phylogeographical history of the alpine shrub *Rhododendron ferrugineum* (Ericaceae) in the Pyrenees. This species is insect pollinated and reproduces both sexually (mixed mating system) and vegetatively through layering. It is widely distributed at high elevation, on non-calcareous bedrock. The aim of the present study is to understand to what extent the phylogeographical history of the species has shaped its present genetic patterns. The Asian origin of the genus *Rhododendron* [27] implies an east/west migration from Asia to Europe. In France, this would lead to the Alps being colonized by *R. ferrugineum* before the Pyrenees, with the Pyrenean populations most likely derived from populations from the Alps. After the migration to the Pyrenees, the species had to survive successive glaciation events. Different hypotheses of glacial refugia and postglacial colonization and their consequences on the genetic patterns of *R. ferrugineum* may be proposed: 1. The species could have survived glaciation episodes in one or both of the two main refugia at the eastern and south central part of the Pyrenees (HYP 1; [24]). Thus, the highest genetic diversity should be found in one or both of these areas and the genetic pattern would be shaped by founder effects i.e. gradual reduction of genomic variability along the colonizing routes from main refugia. This would have occurred because colonization usually involves only a fraction of the genetic diversity present in refugial areas [28]. 2. The species could also have survived in nunataks along the mountain chain (HYP 2). Hypothetically, high elevation nunatak populations would have experienced long periods of isolation, inbreeding and genetic drift [18]. This pattern would lead to a patchy distribution of genetic clusters with low levels of genetic diversity and high population differentiation; 3. A pattern with less differentiated clusters than in the previous hypothesis may be observed if the species had survived in large lowland refugia in which extensive gene flow had occurred. The recolonization of highland areas from lowland refugia could have left small, isolated and marginal rear-edge populations like those currently observed at low altitude along the chain (HYP 3). Indeed, the strong abiotic mountain heterogeneity may have allowed remnant populations to match suitable ecological conditions at intermediate or low altitudes. Moreover, the long life span of the clones [29], [30] could have permitted their survival even with low seedling recruitment. Such rear-edge populations should exhibit more rare alleles and higher among-population genetic diversity than populations recently established from high altitude source populations [15]. These three hypotheses are not exclusive and it is conceivable that the genetic pattern observed may result from the different mechanisms at work successively or simultaneously within the bioclimatic regions of the Pyrenees inducing diverse phylogenetic patterns along the chain.

## Materials and Methods

### Species studied

Sampling of the species of interest was conducted in accordance to national and international guidelines (authorization from the ONF for the populations BetL, BetH, LecL, PraL, PuiH, from the Parc National des Pyrénées for the population TroH, from the Conseil général des Pyrénées-Orientales for population BouH, and no permissions were needed for the other sites).

*Rhododendron ferrugineum* L. (Ericaceae) is an evergreen shrub with a mean height of 70 cm that dominates subalpine landscapes in the Alps and Pyrenees between 1500 to 2200 m a.s.l, on north-to-west facing slopes, sometimes reaching 90–100% of the vegetation cover. It is also found in the Jura Mountains, in the Northern Apennines and in the Apuan Alps [31]. In the Pyrenees, infrequent smaller and isolated populations can be found lower down, between 900–1500 m a.s.l. A single population of some scattered individuals remains at low altitude (1500 m a.s.l.) in the French Jura Mountains. *Rhododendron ferrugineum* is a clonal, self-compatible [32] mass-flowering species (producing until 3000 flowers per m^2^; [33]) pollinated by honeybees and bumblebees [32], [34].

### Study sites and sample collection

**Figure 1:**
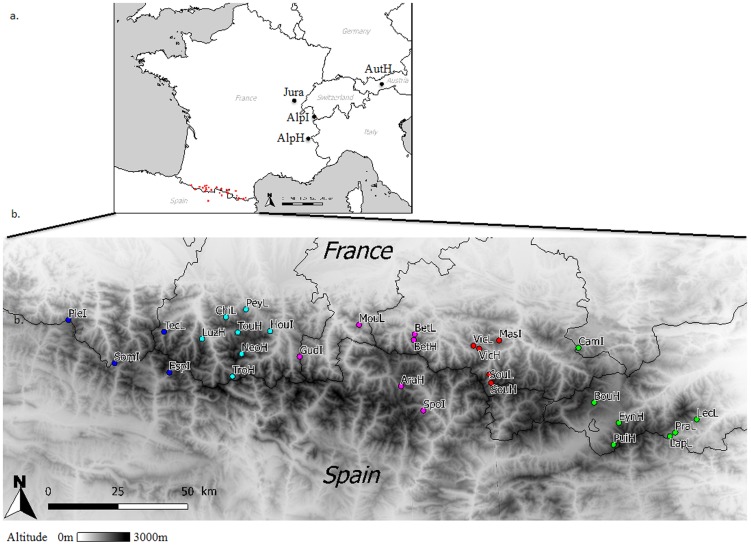
Location of the sampled populations. a. Location of the sampled *Rhododendron ferrugineum* populations in the Alps, Jura Mountains and Pyrenees, b. detailed map of the Pyrenean populations (the colors correspond to the five genetic clusters).

We sampled 29 populations across the Pyrenees, the population in the Jura Mountains and three populations in the Alps (Figure 1, Table 1) ranging from 1070 m to 2200 m a.s.l. The populations were divided into three groups according to their elevation: low altitude (hereafter L; from 1000 to 1450 m a.s.l), intermediate altitude (hereafter I; from 1450 to 1900 m a.s.l.), high altitude (hereafter H; from 1900 to 2200 m a.s.l.). In the Pyrenees, populations were sampled along a longitudinal transect through three regions: Languedoc-Roussillon (eastern part), Midi-Pyrénées (central part) and Aquitaine (western part). Due to calcareous bedrock, populations in the western part of the Pyrenees are rare and we only sampled one. An estimation of population size is given in Table 1 [<1 ha, 1–10 ha, 10–20 ha, 20–50 ha, 50–100 ha, >100 ha). The mean geographical distance between populations from Pyrenees *vs.* Alps, Pyrenees *vs* Jura Mountains and Alps *vs*. Jura Mountains was 900 km (ranging from 515 to 1430 km), 695 km (from 590 to 830 km) and 335 km (from 165 to 640 km) respectively. The distances between populations across the Pyrenees ranged from 3 to 370 km with an average of 126 km. In each of the 33 populations (except for the very small JuraL and LapL populations), young leaves of twenty individuals were sampled (a total of 645 individuals sampled), separated by at least 5 m in order to avoid sampling clonal individuals, were dried in silica gel in the field and then stored at −20°C.

**Table 1:** Geographical information and genetic diversity of *Rhododendron ferrugineum* populations.

Moutain range	Sites	Code population	Location	Population size (ha)	Altitude (m)	n	Ar	Private allele	H_O_	H_E_	F_IS_
Alps	La Vormaine	AlpI	45°02′07″N 06°24′19″E	>100	1560	20	3.70	1	0.49	0.49	**0.04**
	Col du Lautaret	AlpH	46°00′08″N 06°57′18″E	5–10	2100	20	4.30	10	0.59	0.57	**−0.01**
	Parscherkofel	AutH	47°12′32"N 11°27′18"E	50–100	2000	20	2.89	2	0.41	0.39	**−0.03**
Jura		JuraL	42°57′N 05°44′E	<1	1300	10	2.67	0	0.48	0.44	**−0.06**
Eastern Pyrenees	Léca	LecL	42°28′07"N 02°31′40"E	<1	1250	20	2.37	1	0.34	0.30	**−**0.11
	Prats de Mollo	PraL	42°25′21"N 02°24′38"E	<1	1450	20	2.44	1	0.31	0.32	**0.02**
	Lapreste	LapL	42°24′34"N 02°22′51"E	<1	1230	15	2.44	0	0.42	0.34	**−**0.21
	Eyne	EynH	42°28′27"N 02°07′05"E	5–10	1915	20	2.78	0	0.38	0.35	**−0.06**
	Puigmal	PuiH	42°23′26"N 2°05′0"E	5–10	2045	20	2.74	0	0.39	0.34	**−**0.10
	Lac des Bouillouses	BouH	42°33′31"N 01°59′42"E	50–100	2005	20	2.56	0	0.37	0.34	**−0.05**
Central Pyrenees	Camurac	CamI	42°46′30"N 01°55′45"E	10–20	1600	20	2.92	0	0.42	0.38	**−**0.09
	Massat	MasI	42°49′18"N 01°30′75″E	<1	1500	20	2.59	0	0.41	0.40	**0.0007**
	Soulcem	SouH	42°39′28"N 01°27′27"E	50–100	2035	20	2.63	0	0.41	0.39	**−0.01**
	Soulcem	SouL	42°41′28"N 01°27′07"E	<1	1240	20	2.74	0	0.37	0.39	0.08
	Etang de Lhers	VicH	42°47′36"N 01°24′16"E	20–50	1940	20	2.74	0	0.38	0.35	**−**0.07
	Etang de Lhers	VicL	42°48′23"N 01°22′31"E	1–5	1275	20	2.44	0	0.36	0.32	**−**0.09
	Espot	SpoI	42°33′47"N 01°05′36"E	<1	1515	20	4.18	0	0.35	0.43	0.10
	Bethmale	BetL	42°51′42"N 01°04′02"E	1–5	1385	20	2.67	0	0.40	0.37	**−0.06**
	Bethmale	BetH	42°50′24″N 01°03′42"E	50–100	1920	20	2.92	0	0.40	0.37	**−0.03**
	Val d'Aran	AraH	42°39′45"N 00°58′56"E	<1	2075	20	2.26	0	0.29	0.29	**0.05**
	Le Mourtis	MouL	42°54′32″N 00°46′31″E	5–10	1440	20	2.59	0	0.33	0.33	**0.04**
	Peyragudes	GudI	42°47′42"N 00°27′14"E	5–10	1650	20	2.67	0	0.39	0.37	**−0.03**
	Hourquette d'ancizan	HouI	42°53′57″N 00°18′13″E	5–10	1565	20	2.74	0	0.42	0.40	**−0.04**
	Néouvielle	NeoH	42°48′N 00°09′E	50–100	2200	20	2.56	0	0.47	0.44	**−0.03**
	Le Peyras	PeyL	42°59′15"N 00°10′46"E	5–10	1220	20	2.33	0	0.36	0.36	**0.03**
	Col du Tourmalet	TouH	42°53′57″N 00°07′54″E	5–10	1950	20	2.81	1	0.42	0.39	**−0.04**
	Cirque de Troumouse	TroH	42°43′41"N 00°05′40"E	5–10	2080	20	2.44	0	0.39	0.38	**0.02**
	Chiroulet	ChiL	42°57′38"N 00°04′17"E	1–5	1240	20	2.70	0	0.38	0.37	**0.02**
	Luz-Ardiden	LuzH	42°52′45"N 00°03′34"W	10–20	1905	20	2.63	0	0.37	0.37	**0.02**
	Huesca	EspI	42°45′08"N 00°14′18"W	>100	1640	20	2.27	0	0.20	0.30	0.32
	Lac du Tech	TecL	42°54′40"N 00°15′32"W	<1	1240	20	2.41	0	0.39	0.34	**−**0.11
	Col du Somport	SomI	42°47′41″N 00°31′38″W	<1	1645	20	2.26	0	0.33	0.37	0.16
Western Pyrenees	Col de la Pierre Saint Martin	PieI	42°58′07"N 00°45′52″W	<1	1830	20	2.00	0	0.28	0.29	**0.09**

Altitudinal classes are given in the code population (L: low, I: intermediate, H: high altitude).

n  =  sample size, Ar  =  allelic richness, H_O_  =  observed heterozigosity, H_E_  =  expected heterozygosity, F_IS_  =  within population coefficient of inbreeding (non-significant values are in bold, P<0.01).

### DNA extraction and microsatellite markers

For each individual sampled, 20 mg of leaf tissue was ground with two tungsten beads (diameter 3 mm) in a sterile 2 ml Eppendorf tube at 30 Hz for 90 s using TissueLyser II (Qiagen). Then, the quality and amount of DNA obtained from the DNeasy-Plant Mini Kit (Qiagen, Courtaboeuf, France) following the manufacturer's instructions was verified using a Nanodrop ND 1000 spectrophotometer (Peqlab, Erlangen, Germany).

We used nuclear microsatellites as suitable molecular markers to reconstruct regional phylogeographical patterns [18]. Chloroplasts (personal observation) or other types of genetic markers such as AFLP proved to be little informative in this species [35]. The 27 polymorphic microsatellite markers (Table S1) were previously developed [36], [37] using pyrosequencing technologies (454 FLX Titanium, Roche Applied Science, Meylan, France). Forward primers were labeled with fluorochromes (Eurofins MGW Operon, Courtaboeuf, France) and were used in six multiplexed PCRs, optimized using Multiplex Manager 1.1 [38].

Amplifications were carried out in a 10 μl mix containing 10 ng of template DNA, 0.7 × Qiagen Multiplex PCR Master Mix, 10 μM of each primer and RNase free water. Cycling conditions were 15 min at 95°C, 30 × (30 s at 95°C, 90 s at 56°C, 45 s at 72°C) and 30 min at 60°C. Each set of reactions included a negative (water) and a positive (known genotype) control. Fragment lengths were read on an ABI 3730 sequencer (Applied Biosystems, Courtaboeuf, France) and scored with Genemapper version 4.0 software with the GeneScan – 600 LIZ (Applied Biosystems, Courtaboeuf, France) as internal size standard.

### Data analysis

#### Genetic diversity

All 645 individuals were tested for clonality with GenAlEx 6.5 software [39]. The presence of null alleles at each locus was examined using Micro-Checker software [40], and tests for departures from Hardy-Weinberg equilibrium (HWE) were tested by Fisher's exact tests using Genepop 3.4 [41]. We used GenAlEx 6.5 software to calculate for each locus and population the number of alleles, the allelic richness (Ar), the observed (H_O_) and expected heterozygosity (H_E_) and the inbreeding coefficient (F_IS_).

#### Population structure and differentiation

The pairwise F_ST_ (level of genetic differentiation; [42]) among populations were calculated with Genodive v2.0 [43]. Then, the statistical significance of the pairwise F_ST_ values was assessed by 1000 random permutations of individuals across populations with Genetix 4.05 [44].

To analyze the genetic structure among populations, we used Structure software (ver. 2.3.3; [45]) in order to determine the most likely number of homogenous clusters in the sample (K). The admixture model was applied with 50 runs for each K value from 1 to “number of populations +1”, each run comprising a burn-in period of 20 000 iterations followed by 100 000 iterations. The optimal value of *K* was evaluated by considering the highest mean likelihood value of *K*, *i.e.*, L (*K*), as well as the Δ*K* method [46]. These results were computed and visualized in the online interface Structure Harvester [47].

To evaluate the distribution of genetic variance within and among populations, and among groups when populations were nested into genetic clusters, altitude (low, intermediate and high altitude) or population size (according to the six population size groups in Table 1) we performed analyses of molecular variance (AMOVA; [48]) using Genodive v2.0. The significance of the results was tested using 999 random permutations of the data.

Mantel tests [49] were performed (“ncf” package implemented in R, R Development Core Team, 2008) to test for isolation-by-distance (IBD; divergence due to drift and mutation proportionally increasing with geographical distance), investigating the correlation between matrices of genetic (*F*_ST_) and geographical (km) distances. Mantel test were also used to determine wheter elevational separation was related to genetic differentiation. Calculation of the gene flow on the basis of rare alleles was conducted [50] and the number of migrants (N_m_) was corrected for the number of samples.

## Results

A total of 153 alleles at 27 microsatellite loci were detected across 645 individuals at all sites (Table S2). No clones were identified among the individuals sampled as revealed by the test of clonality.

Significant departures from HWE across loci were detected in 12 out of 33 (or 36%) sites. The RF87P2 marker was the only locus that showed no departures from HWE. Most of the loci showed significant departures from HWE in two to seven populations, except RF14P3, RF47P1 (11 populations each), RF56P1 (13 populations) and RF96P2 (31 populations). These instances likely reflect occasional departures from random mating rather than the presence of null alleles. Indeed, the probability of null alleles was low for all loci, according to Oosterhout's method [40], mean estimation of null alleles was −0.031 (ranging from −0.3675 to 0.1352). No differences in the results were observed when including the microsatellite marker RF96P2 or not, so we decided to keep it in the analyses.

### Genetic diversity and allelic richness among populations

The number of alleles ranged from 2 to 15 (mean 6.25) alleles per locus. A total of 16 private alleles (restricted to a single population; Table 1) was detected (10.45%), mostly in AlpH (10), AutH (2), AlpI (1) and also in some Pyrenean populations (TouH, PraL, LapL had one private allele each). Allelic richness (Ar) varied across sites (2–4.30). The highest Ar were observed in the Alps (AlpI = 3.70 and AlpH = 4.30) and in one of the southernmost populations of the central Pyrenees (SpoI = 4.18), while the lowest Ar were observed at the western end of the Pyrenees (PieI = 2.00, SomI = 2.26, EspI = 2.27) and in the central Pyrenees (AraH = 2.26). Ar, H_O_ and H_E_ were 4.6, 0.37 and 0.35 respectively in the Pyrenees, and 5.2, 0.5 and 0.45 in the Alps. Expected (H_E_) and observed heterozygosity (H_O_) ranged from 0.20 to 0.59 and from 0.29 to 0.56 respectively for all sites. H_E_ was high in the Alps and Jura Mountains (ranging from 0.39 to 0.57), and in some Pyrenean populations (SpoI and NeoH, 0.43 and 0.44 respectively) and the lowest in the easternmost (LecL, PraL, AraH) and westernmost (EspI, PieI) Pyrenean populations. These latter populations were small and isolated (<1 ha) except the large and central population EspI (>100 ha). Significant heterozygosity excesses (F_IS_ coefficients ranging from −0.07 to −0.21) were detected in three eastern (LecL, LapL, PuiH) and four central populations of the Pyrenees (CamI, VicH, VicL, TecL). In contrast, significant heterozygosity deficiency was found in four populations with particularly high values of F_IS_ coefficients in SomI (0.16) and EspI (0.31). Because the frequencies of null alleles did not appear to be significant, the highest F_IS_ values could be due to inbreeding.

### Genetic differentiation and structuration among populations

The F_ST_ values (Table S3) were significantly different from zero for all population pairs, except for three pairwise comparisons: BetH/BetL and SouH/SouL that were geographically really close (less than 3 km) and CamI/EynH (35 km apart). The mean F_ST_ value was relatively low (0.267) and most of the highest values (>0.33) were found in the Pyrenees between eastern and western populations, reaching 0.45 for LecL/AraH. The population from the Jura Mountains showed a highly variable level of F_ST_ (from 0.25 to 0.42).

**Figure 2:**
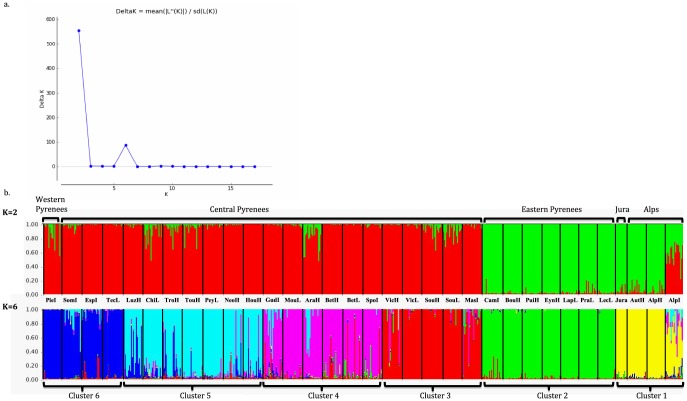
Genetic structure of the 33 populations. a. Plot of Delta K according to K. b. Structure clustering results obtained at K = 2 and K = 6. Each individual is represented by a thin bar corresponding to the sum of assignment probabilities to the K cluster. Black bars separate populations.

To infer the relationship between populations we used the clustering algorithm implemented in the program Structure. Comparing all 33 populations (K: 1 to 34), ΔK as a function of K reached a peak at K = 2 and then reached a second peak at K = 6 (Figure 2a). At K = 2, populations from the Alps, Jura and the eastern Pyrenees were assigned to the same cluster while populations from western and central Pyrenees were clearly grouped apart. Individuals from AlpI showed a similar proportion of membership to both genetic clusters. At K = 6, populations from the Alps and Jura were assigned to one cluster (CL1) and those of the Pyrenees were grouped in 5 clusters (CL2 to CL6) along a longitudinal gradient. Except between CL5 and CL4 or CL6, there was low admixture between the other Pyrenean clusters.

The analysis of molecular variance (AMOVA; Table 2) based on F_ST_ showed that as expected for polymorphic loci such as microsatellites, the within-population variance component was rather high (>66%, P = 0.001). The among variation component (F_ST_) ranged from 9% to 31% according to the grouping and was statistically significant in all combinations tested (P = 0.001). At a superior hierarchical level, the genetic clusters could explain a significant part of the total genetic variance (24.4%, P = 0.001) whereas it was not significant when grouping populations into altitudinal groups (P = 0.956) or population size (P = 0.222).

**Table 2:** Partitioning of the observed microsatellite variation (AMOVA) based on F_ST_ of *Rhododendron ferrugineum* populations.

Source of Variation	SSD	d.f.	MS	Var-comp	% Variation	P-value
Within Population	4355.120	548	7.947	7.947	0.662	0.001
Among Population	732.618	24	30.526	1.135	0.095	0.001
Among Genetic cluster	1461.242	4	365.310	2.929	0.244	0.001
Within Population	4354.038	548	7.945	7.945	0.690	0.001
Among Population	2103.864	26	80.918	3.659	0.310	0.001
Among Altitude	93.988	2	46.994	−0.167	0.00	0.956
Within Population	4366.874	548	7.894	7.894	0.685	0.001
Among Population	2079.120	23	83.837	3.372	0.293	0.001
Among Population size	106.877	5	56.922	0.258	0.022	0.222

Allelic richness (Ar) was highest in CL1 (Alps and Jura, 5.2) and lowest in CL6 (western Pyrenees, 2.9) and ranged from 3.2 to 3.8 in the other clusters (Table 3). F_ST_ values were all significantly different among clusters (Table 4). The highest F_ST_ values were found between CL2 (eastern Pyrenees) and CL5 or CL6 (western Pyrenees, 0.29 and 0.31 respectively). The F_ST_ values between CL1 and the Pyrenean clusters were quite low (ranging from 0.16 to 0.20).

**Table 3:** Genetic diversity of *Rhododendron ferrugineum* per genetic cluster.

	Ar	H_O_	H_E_	F_IS_
Cluster 1	5.630	0.498	0.584	0.148
Cluster 2	3.630	0.375	0.364	−0.030
Cluster 3	3.185	0.386	0.406	0.048
Cluster 4	3.778	0.360	0.390	0.078
Cluster 5	3.481	0.402	0.429	0.062
Cluster 6	2.926	0.315	0.384	0.179

Ar  =  allelic richness, H_O_  =  observed heterozigosity, H_E_  =  expected heterozygosity, F_IS_  =  within population coefficient of inbreeding.

**Table 4:** F_ST_ values among genetic clusters (p-values for all pairs<0.001).

	Cluster 1	Cluster 2	Cluster 3	Cluster 4	Cluster 5	Cluster 6
Cluster 1	—	0.203	0.171	0.177	0.165	0.166
Cluster 2		—	0.237	0.275	0.297	0.311
Cluster 3			—	0.101	0.153	0.230
Cluster 4				—	0.108	0.235
Cluster 5					—	0.129
Cluster 6						—

There was a positive relationship (Mantel test) between genetic distance and geographical distance matrices across populations located in the Pyrenees (R = 0.784, P = 0.001). The increase of isolation with geographical distance was consistent with the previous results (see Structure analyses). When including populations from the Alps and Jura, the Mantel test was still positive (R = 0.26) but not significant (P = 0.383), confirming the lack of strong differentiation among the populations from the Alps and Pyrenees. At the Pyrenean scale, the correlation among elevational distances and FST was slightly positive but not significant (R = 0.092, P = 0.06).

### Gene flow among populations

Gene flow among all studied populations (Alps, Pyrenees and Jura) was low and reached a mean value of Nm = 0.72 migrants per generation. The values reached 2.88 for all Pyrenean populations but 2.10 in the eastern lineage (CL2) and 5.9 in the western lineage (CL3 to CL6).

## Discussion

### Genetic diversity in R. ferrugineum populations

Microsatellite analysis revealed a relatively high level of genetic variability in *R. ferrugineum* populations in the Pyrenees (Ar: 2–4.18, H_E_: 0.29–0.44) but lower than in the Alps and the Apennine populations (Ar: 2.71–5.57, H_E_: 0.37–0.68; [31]) or in other long-lived species found in the Pyrenees (*Pinus uncinata*: H_E_: 0.83–0.99, (25); *Quercus petraea*: H_E_: 0.76–0.84, [51]). Although clonality and selfing are known to decrease genetic variability [52], the long life span of individuals [29], [30] and the strong life-time inbreeding depression eliminating most selfed individuals (personal data) may contribute to maintaining relatively high genetic diversity even in small *R. ferrugineum* populations. Accordingly, most of the populations studied showed a F_IS_ close to zero and a great proportion of the genetic variation was found within populations as observed in other *R. ferrugineum* populations [31] and other alpine species [19], [53], [18]. However, selfing has probably contributed to individual recruitment in populations harboring higher heterozygote deficiency (SomI, PieI, EspI, SpoI). Such high heterozygote deficiency has also been reported in the Apennines and the Alps [31]. Surprisingly, significant heterozygote excess occurred mostly in eastern (LapL, LecL, PuiH), and central populations (CamI, VicH, VicL) and in one of the westernmost populations (TecL).

### Alpine origin of the two Pyrenean *R. ferrugineum* lineages

The *Rhododendron* genus is known to originate from Asia [27] and colonized Europe from east to west. Our hypothesis was that migration from the Alps to the Pyrenees occurred with a loss of genetic diversity along the colonization routes. In our study, we identified two lineages in the Alps: the first, found only in the northern population (AlpI), was related to western Pyrenean populations. This lineage would possibly be found in more populations with more extensive sampling in the Alps. The second lineage, related to the eastern and central Pyrenees, was found in the same population AlpI but also in south and central-eastern populations from the Alps and in the Jura Mountains. At K = 6, populations from the Jura Mountains and the Alps are found in the same cluster, that may indicate the occurrence of recent gene flow between the Alps and the Jura Mountains, possibly in low land refugia during glaciations. It would be interesting to complete our data by a large sampling of populations from the Alps and the Jura Mountains in order to detail the European phylogenetic pattern of the species. Compared to the Alpine populations, Pyrenean populations were characterized by a substantial loss of genetic diversity (Ar, H_E_, H_O_) suggesting bottlenecks during the migration events [54], [16]. Cold, dry steppe/tundra occupied most of the land between the Alps and the Pyrenees during the last maximum glaciation [55] and could have allowed migration of Alpine species such as *R. ferrugineum* either from the southwestern Alps [56], [57] or from northern Alpine populations through the French Massif Central which is considered to be a key area in the colonization of the Pyrenees by Alpine species [58].

### Genetic pattern and phylogeographical history of *R. ferrugineum* in the Pyrenees

We found that genetic diversity of *R. ferrugineum* was not correlated to altitude or population size; instead there was a sharp longitudinal split between the two genetic lineages. Other studies in alpine ecosystems similarly found no changes in genetic diversity depending on population size or along altitudinal gradients [59]. Along environmental gradients, several parameters such as overlap of generations or recruitment frequency may influence intrapopulation genetic variation and gene flow among populations may be sufficient to maintain relatively high genetic diversity even in the smallest populations.

The longitudinal genetic pattern seemed to result from the migration of two Alpine lineages and from the establishment of populations in refugia along the Pyrenean chain due to glaciation events. The strong differentiation between the eastern and western Pyrenean lineages and the low level of gene flow observed between the two suggested that very few admixtures occurred since the two lineages had become established. This may result from topographical barriers (high summits, glaciated areas), seed dispersion by gravity and short pollinator travels hampering gene flow among populations and genetic homogenization. An east-west genetic separation had also been observed in other Pyrenean species (i.e. *Papaver alpinum,* [57], *Trifolium alpinum*, [19]). The populations of the eastern lineage were little differentiated even though they were distant from one another (35 km between the two undifferentiated populations CamI and EynH), were of very different sizes, grew in contrasting environments, and experienced a low level of among-population current gene flow. Very small and isolated populations (PraL, LecL, LapL) showed a relatively high level of intrapopulation heterozygosity (H_E_, H_O_) and exhibited private alleles (PraL, LapL) despite relatively lower allelic richness likely due to the loss of rare alleles by genetic drift, common in such small populations [60]. These populations survive in rather unusual ecological conditions for this species, i.e. on north facing slopes in deep, shaded valleys and mainly beneath beech forest (*Fagus sylvatica*) or sometimes in the vicinity of Mediterranean *Quercus ilex* forests. These data suggested that they were more likely to be rear-edge remnant populations [12] left there after the recolonization of highland areas (HYP 3) rather than the result of a secondary colonization of lowland areas by high altitude individuals. Together, these findings and the absence of clear founder effects suggested that the eastern populations within CL2 have experienced recent and extensive gene flow. Such intense gene exchanges could have occurred in large lowland refugia during the cold periods of the Pleistocene and possibly later in genetically connected populations growing over high altitude plateaus, which are typical of eastern Pyrenees landscapes. These large high altitude populations could have then become fragmented possibly during the last 30 ky when climate became warmer and drier [26].

The four well separated and differentiated genetic clusters nested in the western Pyrenean lineage appeared as a sub-structure of the east/west configuration and presumably diverged more recently. Overall, we did not observe a gradient of genetic variability loss among these clusters as expected from a range-expansion wave [11]. Rather, we observed a consistent genetic heterozygosity and allelic richness and a significant isolation by distance (Mantel test) among them. Wright's isolation by distance model suggests that gene flow is reduced as geographical distance increases. This may explain the very low level of admixture between CL3/CL6. The patchy distribution of often homogenous and highly distinct clusters enclosing a similar level of within- and between-population genomic variability is compatible with the hypothesis of the survival of the species in numerous Pyrenean refugia as proposed for other Pyrenean species [19],[18]. Moreover, the level of differentiation, the sharp genetic boundaries between most clusters and the number of migrants per generation suggested a long isolation of populations and the action of genetic drift in several high altitude refugia (HYP 2) rather than the survival in large and genetically connected populations in low elevation unglaciated areas. The higher genetic diversity in SpoI likely accounts for such refugia as well as TouH (having one private allele) where genetic variability was greater than in other populations. However, the relatively higher level of admixture between CL5 and CL4 or CL6 suggested that they had recently exchanged genes. Because the admixture concerned mainly isolated and sometimes very distant (AraH and CL5 populations) populations at high and intermediate altitude, contemporary gene exchanges among them through pollen or seed dispersal was very unlikely. We suspected that, at least in this part of the Pyrenees, longitudinal gene exchanges could have occurred possibly in lowland refugia across Pyrenean piedmonts before the post-glacial recolonization of high altitude area through valleys (HYP 1). Such a recolonizing pattern has been observed in sessile oak [51] and suggested for *Aster pyrenaeus* [20]. Thus, the phylogeographical pattern in central and western Pyrenees could result both from population isolation and differentiation in nunataks and gene exchange in some lowland refugia. Among clusters, CL6 showed the lowest genetic variability (EspI, SomI and PieI) and genetic diversity gradually decreasing in this cluster along the sequence TecL, SomI and PieI. Thus, the genetic pattern of CL6 could be chiefly related to founder events and the loss of genetic diversity and heterozygosity [11] during westward expansion of the species.

### Implications for conservation

The longitudinal genetic structuration found in *R. ferrugineum* and particularly the east/west separation between the two main genetic lineages has already been observed in other studies [57], [19]. This suggests that many other alpine species may have been subject to similar events as *R. ferrugineum* and, nowadays, could present a comparable genetic pattern. We state that these data now should be usefully considered in management projects for Pyrenean species particularly those having an extended east/west distribution. According to the European Economic Community laws (92/43/EEC habitat directives, 1992) *R. ferrugineum* heathlands must be locally preserved due to their high ecological and patrimonial value. We think that, henceforth, in aim to preserve genetic diversity of the species, the genetic pattern here revealed should be taken into consideration to choose sites that have to be protected. The eastern lowland isolated populations (LecL, PraL, LapL) grow in very specific habitat and present rare alleles. According to the “rear edge population hypothesis”, they harbor all together the bulk of species' genetic diversity [12]. Scientific programs have to be developed to follow the population dynamic and the genetic diversity evolution of these very original populations and take an inventory of other currently unknown rear-edge populations. Moreover, studying population dynamics at the edge of a species' range is crucial to understand the response of species to environmental changes and their susceptibility to extinction [31]. Also the populations TouH (presenting a rare allele) and SpoI (having a high allelic richness) should be considered as populations of interest for conservation. However, other parameters should be taken into account such as surrounding plant community or pollinator diversity. For example, it is known that the number of bumblebees species, which are one of the main pollinators of *R. ferrugineum*, is high in the eastern Pyrenees, especially close to population EynH [61]. Consequently, it could be an interesting population to monitor.

In conclusion, the Pyrenees are usually considered to only be a latitudinal barrier to northward or southward species expansions. In agreement with previous findings [18], [19], our study of the subalpine shrub *R. ferrugineum* highlights a much more complex role of the Pyrenees in species genetic pattern and biogeography. The species showed two genetically isolated lineages, which have probably colonized the Pyrenees from the Alps and Jura Mountains. Our findings suggest that the eastern lineage most likely survived in large lowland refugia and that low-altitude small and isolated rear-edge populations have survived until now after the upward recolonization of highland areas agreeing with our HYP 3. Extensive gene flow may have occurred in lowland refugia as well as among populations growing in high altitude plateaus. The sub-structure of the central and western lineage suggests that the species mostly survived glaciations in isolated nunataks (HYP 2) but that some lowland refugia could also have played a role locally (HYP 1). Our study provides a useful genetic reference framework to select *R. ferrugineum* heathlands in management projects in the Pyrenees and points out priority populations for protection.

## Supporting Information

Table S1 Characteristics of 27 polymorphic microsatellite markers in *Rhododendron ferrugineum*.
(DOCX)

Table S2 Genetic parameters for the 27 microsatellite markers (non-significant values are in bold, P<0.01).
(DOCX)

Table S3 Pairwise F_ST_ comparisons between all sampling sites above the diagonal as calculated by Genetix. Non-significant F_ST_ are in bold.
(DOCX)
