# Supplementary material for: Microsatellite Marker Analysis Reveals the Complex Phylogeographic History of Rhododendron ferrugineum (Ericaceae) in the Pyrenees
Source: PLoS One. 2014 Mar 25;9(3):e92976. doi: 10.1371/journal.pone.0092976 (PMC3965482; doi:10.1371/journal.pone.0092976)
Supplement: Table S1 — Characteristics of 27 polymorphic microsatellite markers in Rhododendron ferrugineum. (DOCX) [file pone.0092976.s001.docx]

# Supporting Information

Table S1. Characteristics of 27 polymorphic microsatellite markers in *Rhododendron ferrugineum*.

|  | | | | |  |  |
| --- | --- | --- | --- | --- | --- | --- |
| Locus | GenBank accession number | Repeat motif | Allele size range (bp) | Primer sequences (5'-3') | Multiplex marker set | Fluorolabel |
| RF96P2 | KC008581 | (TCT)_9_ | 73-79 | F : AGGCCTTCTACTACAACAACTCA | 1 | Yakima Yellow |
|  |  |  |  | R : TCCACCCTTTCCTTCTCTTTC |  |  |
| RF85 | KC008582 | (CAA)_5_ | 148-159 | F : TGGTGCTGTGACAAAGGGTA | 1 | Atto550 |
|  |  |  |  | R : TCCGAGGAAATCAATGAAGG |  |  |
| RF153 | KC008583 | (GA)_7_ | 207-235 | F : CCACACGCTAGGGAACTTTT | 1 | Fam |
|  |  |  |  | R : TCAGCGTCGAAGAATCTCAA |  |  |
| RF140 | KC008584 | (AC)_13_ | 196-208 | F : ATGGCTAGCTTTGTGCTGCT | 1 | Yakima Yellow |
|  |  |  |  | R : TGCACATGAGTTCCTCAACA |  |  |
| RF175 | KC008585 | (TG)_10_ | 282-318 | F : TTCTGAACCCTGCACTTCCT | 1 | Atto565 |
|  |  |  |  | R : CCAGCGGAGACAGTATGGAT |  |  |
| RF213 | KC008586 | (AC)_9_ | 87-113 | F : CGTCTAAGAAAGTCGCACCA | 2 | Atto565 |
|  |  |  |  | R : GAAGGGTTGAAGGATGACGA |  |  |
| RF163P1 | KC008587 | (GA)_12_ | 179-191 | F : TGAATGGTTCAAGCACCAAA | 2 | Fam |
|  |  |  |  | R : GCTGTTCTTGCTGACATGGA |  |  |
| RF122P2 | KC008588 | (TTC)_7_ | 209-238 | F : TTCTCAGTTCCTTCTTCGGC | 2 | Yakima Yellow |
|  |  |  |  | R : GTAAGAGAAAAGGACGCGCA |  |  |
| RF113P2 | KC008589 | (GTT)_8_ | 312-327 | F : TCGTCAAATGCAGCCAAAC | 2 | Atto550 |
|  |  |  |  | R : CAACGAACAATCATGCTCAAA |  |  |
| RF202 | KC008590 | (TG)_15_ | 86-104 | F : CATTTTCCAGCACAAAACTAAATG | 3 | Atto565 |
|  |  |  |  | R : TGTGACGGTGTATCGGAAGA |  |  |
| RF128 | KC008591 | (GA)_11_ | 146-156 | F : ATAAACGGCTCTCAAATGCG | 3 | Fam |
|  |  |  |  | R : GATGTGTTTCCGGCGTAGTT |  |  |
| RF157P1 | KC008592 | (TC_)12_ | 200-216 | F : CTTCCTCTCCCATGCCATAA | 3 | Atto550 |
|  |  |  |  | R : CCTTTCCGAAGCACCAATAA |  |  |
| RF105P2 | KC008593 | (GAA)_6_ | 460-466 | F : GACTTGCTCACGGGAACCTA | 3 | Yakima Yellow |
|  |  |  |  | R : ATTGGATCATGGGTCAAAGC |  |  |
| RF87P2 | KC008594 | (CAC)_7_ | 133-142 | F : CCTTTCCTCGCAATGAAGAA | 4 | Fam |
|  |  |  |  | R : AGGAAGGTGATGAGGAGGGT |  |  |
| RF182 | KC008595 | (CT)_12_ | 150-164 | F : TCTGTCCGACCGAATCTGTA | 4 | Yakima Yellow |
|  |  |  |  | R : CAGCAGCCATTAGACAGAAAAA |  |  |
| RF146P1 | KC008596 | (TC)_10_ | 211-261 | F : CCCCTTAAATGGGAGGCTTA | 4 | Atto550 |
|  |  |  |  | R : AAACTCCAAGGGCATGTTTG |  |  |
| RF114P3 | KC008597 | (CTT)_7_ | 385-394 | F : TGCACTAACCCAGAAATCATGT | 4 | Atto565 |
|  |  |  |  | R : TCATAATCGGGTTTCAGCTA |  |  |
| RF126P1 | KC008598 | (TG)_9_ | 438-450 | F : AATTAACCATCAACCGTAGGGTAA | 4 | Atto550 |
|  |  |  |  | R : TCAAACAACTTCATCCCTTCC |  |  |
| RF47P1 | HQ822280 | (TC)_12_ | 59-83 | F : CTCCTCGTCGTCACACATTC | 5 | Fam |
|  |  |  |  | R : CCATGCGGCTTGATAGGTAG |  |  |
| RF14P3 | HQ822277 | (GGA)_5_ | 332-338 | F : ACATAGAGCATTTGGCCGAC | 5 | Fam |
|  |  |  |  | R : GGAGTCGAGTGGTGTTCGTT |  |  |
| RF38P1 | HQ822281 | (TCC)_4_ | 226-235 | F : TTCAATCGGACATTATTACCTTG | 5 | Atto565 |
|  |  |  |  | R : AGGATGAGCTGTGAGTCGGT |  |  |
| RF41P1 | HQ822282 | (TC)_9_ | 184-234 | F : TCCAAGTGGTTATGTAATTCTATCG | 5 | Atto550 |
|  |  |  |  | R : CTATGCTTCGGCAAAAGGAA |  |  |
| RF46P2 | HQ822276 | (CTC)_6_ | 161-164 | F : CAGAGGCCTACACTTCTGCC | 6 | Fam |
|  |  |  |  | R : ACAGGAAGGTTAGGAGGGGA |  |  |
| RF6P2 | HQ822279 | (TCTG)_4_ | 208-228 | F : CAGCGTGATAAATTGAGGGG | 6 | Yakima Yellow |
|  |  |  |  | R : TACACCGTTCTGCCATTCAG |  |  |
| RF56P1 | HQ822283 | (AC)_6_ | 73-85 | F : AATGTATTTATTGTCTTTATCCCCA | 6 | Yakima Yellow |
|  |  |  |  | R : CTTGAGGTGTGCAGCTTTGA |  |  |
| RF74P1 | HQ822278 | (GA)_13_ | 152-182 | F : ATGCCACCAAATCTATTGCC | 6 | Atto565 |
|  |  |  |  | R : TTTCTCTCCTGCACGCTTCT |  |  |
| RF81P1 | HQ822275 | (GA)_11_ | 140-154 | F : CGAAGGACCAACTGAAAAGG | 5 | Yakima Yellow |
|  |  |  |  | R : ACCATGGCCACTGTTTTACC |  |  |
