# Supplementary material for: Microsatellite Marker Analysis Reveals the Complex Phylogeographic History of Rhododendron ferrugineum (Ericaceae) in the Pyrenees
Source: PLoS One. 2014 Mar 25;9(3):e92976. doi: 10.1371/journal.pone.0092976 (PMC3965482; doi:10.1371/journal.pone.0092976)
Supplement: Table S2 — Genetic parameters for the 27 microsatellite markers (non-significant values are in bold, P<0.01). (DOCX) [file pone.0092976.s002.docx]

Table S2. Genetic parameters for the 27 microsatellite markers (non-significant values are in bold, P<0.01)

|  | | | |  |
| --- | --- | --- | --- | --- |
| **Locus** | **Number of alleles** | **H_O_** | **H_E_** | **F_IS_** |
| **Rf113P2** | 5 | 0.877 | 0.796 | -0.121 |
| **Rf122P2** | 7 | 0.680 | 0.609 | -0.125 |
| **Rf163P1** | 6 | 0.858 | 0.809 | -0.093 |
| **Rf213** | 7 | 0.665 | 0.693 | **-0.022** |
| **Rf105P2** | 3 | 0.873 | 0.775 | -0.133 |
| **Rf128** | 5 | 0.883 | 0.805 | -0.115 |
| **Rf157P1** | 8 | 0.850 | 0.781 | -0.110 |
| **Rf202** | 6 | 0.851 | 0.845 | -0.085 |
| **Rf85** | 4 | 0.813 | 0.777 | **-0.054** |
| **Rf87P2** | 4 | 0.858 | 0.794 | -0.106 |
| **Rf96P2** | 2 | 0.685 | 0.728 | **0.032** |
| **Rf114P3** | 4 | 0.708 | 0.633 | -0.132 |
| **Rf126P1** | 3 | 0.678 | 0.595 | -0.137 |
| **Rf140** | 5 | 0.824 | 0.848 | **-0.002** |
| **Rf146P1** | 10 | 0.864 | 0.789 | -0.110 |
| **Rf153** | 9 | 0.825 | 0.811 | **-0.035** |
| **Rf175** | 6 | 0.873 | 0.827 | -0.087 |
| **Rf182** | 5 | 0.871 | 0.779 | -0.133 |
| **Rf6P2** | 4 | 0.778 | 0.702 | -0.123 |
| **Rf14P3** | 3 | 0.669 | 0.591 | -0.141 |
| **Rf38P1** | 3 | 0.686 | 0.602 | -0.136 |
| **Rf41P1** | 8 | 0.844 | 0.831 | **-0.041** |
| **Rf46P2** | 2 | 0.825 | 0.728 | -0.139 |
| **Rf47P1** | 10 | 0.809 | 0.796 | -0.078 |
| **Rf56P1** | 3 | 0.845 | 0.757 | -0.132 |
| **Rf74P1** | 15 | 0.902 | 0.835 | -0.101 |
| **Rf81P** | 6 | 0.864 | 0.777 | -0.119 |
