# Supplementary material for: Microsatellite Marker Analysis Reveals the Complex Phylogeographic History of Rhododendron ferrugineum (Ericaceae) in the Pyrenees
Source: PLoS One. 2014 Mar 25;9(3):e92976. doi: 10.1371/journal.pone.0092976 (PMC3965482; doi:10.1371/journal.pone.0092976)
Supplement: Table S3 — Pairwise FST comparisons between all sampling sites above the diagonal as calculated by Genetix. Non-significant FST are in bold. (DOCX) [file pone.0092976.s003.docx]

Table S3. Pairwise F_ST_ comparisons between all sampling sites above the diagonal as calculated by Genetix. Non-significant F_ST_ are in bold.

| **Statistic** | **AlpH** | **AutH** | **Jura** | **LecL** | **PraL** | **LapL** | **EynH** | **PuiH** | **BouH** | **CamI** | **MasI** | **SouL** | **SouH** | **VicL** | **VicH** | **SpoI** | **BetL** | **BetH** | **AraH** | **MouL** | **GudI** | **HouH** | **EspI** | **NeoB** | **PeyL** | **TouH** | **TroH** | **ChiL** | **LuzH** | **TecL** | **SomI** | **PieI** |
| --- | --- | --- | --- | --- | --- | --- | --- | --- | --- | --- | --- | --- | --- | --- | --- | --- | --- | --- | --- | --- | --- | --- | --- | --- | --- | --- | --- | --- | --- | --- | --- | --- |
| **AlpI** | 0.141 | 0.241 | 0.139 | 0.289 | 0.279 | 0.258 | 0.255 | 0.263 | 0.257 | 0.225 | 0.178 | 0.214 | 0.210 | 0.262 | 0.239 | 0.172 | 0.164 | 0.178 | 0.199 | 0.229 | 0.173 | 0.157 | 0.219 | 0.140 | 0.216 | 0.171 | 0.186 | 0.151 | 0.173 | 0.174 | 0.201 | 0.261 |
| **AlpH** | -- | 0.240 | 0.118 | 0.255 | 0.272 | 0.232 | 0.247 | 0.252 | 0.240 | 0.219 | 0.217 | 0.242 | 0.235 | 0.283 | 0.261 | 0.238 | 0.233 | 0.236 | 0.295 | 0.289 | 0.237 | 0.247 | 0.297 | 0.229 | 0.306 | 0.251 | 0.251 | 0.269 | 0.281 | 0.236 | 0.257 | 0.325 |
| **AutH** | -- | -- | 0.341 | 0.377 | 0.393 | 0.361 | 0.348 | 0.335 | 0.348 | 0.313 | 0.308 | 0.293 | 0.311 | 0.389 | 0.357 | 0.340 | 0.345 | 0.357 | 0.408 | 0.392 | 0.324 | 0.331 | 0.372 | 0.299 | 0.364 | 0.331 | 0.330 | 0.308 | 0.319 | 0.300 | 0.266 | 0.346 |
| **Jura** | -- | -- | -- | 0.318 | 0.325 | 0.287 | 0.283 | 0.307 | 0.293 | 0.263 | 0.256 | 0.281 | 0.273 | 0.345 | 0.327 | 0.292 | 0.286 | 0.288 | 0.351 | 0.367 | 0.304 | 0.315 | 0.363 | 0.291 | 0.360 | 0.326 | 0.299 | 0.310 | 0.329 | 0.326 | 0.328 | 0.421 |
| **LecL** | -- | -- | -- | -- | 0.133 | 0.104 | 0.063 | 0.066 | 0.064 | 0.062 | 0.283 | 0.339 | 0.303 | 0.394 | 0.373 | 0.330 | 0.305 | 0.328 | 0.450 | 0.421 | 0.356 | 0.377 | 0.444 | 0.353 | 0.429 | 0.396 | 0.376 | 0.388 | 0.397 | 0.379 | 0.389 | 0.446 |
| **PraL** | -- | -- | -- | -- | -- | 0.044 | 0.073 | 0.094 | 0.072 | 0.090 | 0.235 | 0.313 | 0.283 | 0.365 | 0.351 | 0.309 | 0.265 | 0.287 | 0.425 | 0.395 | 0.326 | 0.353 | 0.427 | 0.328 | 0.401 | 0.362 | 0.354 | 0.340 | 0.379 | 0.373 | 0.352 | 0.411 |
| **LapL** | -- | -- | -- | -- | -- | -- | 0.051 | 0.085 | 0.042 | 0.054 | 0.191 | 0.253 | 0.222 | 0.316 | 0.301 | 0.262 | 0.237 | 0.249 | 0.382 | 0.359 | 0.288 | 0.326 | 0.411 | 0.303 | 0.380 | 0.339 | 0.319 | 0.323 | 0.361 | 0.361 | 0.347 | 0.416 |
| **EynH** | -- | -- | -- | -- | -- | -- | -- | 0.034 | 0.013 | **0.011** | 0.217 | 0.265 | 0.233 | 0.329 | 0.309 | 0.276 | 0.260 | 0.285 | 0.402 | 0.377 | 0.314 | 0.324 | 0.391 | 0.300 | 0.377 | 0.348 | 0.325 | 0.339 | 0.345 | 0.343 | 0.333 | 0.395 |
| **PuiH** | -- | -- | -- | -- | -- | -- | -- | -- | 0.025 | 0.023 | 0.206 | 0.273 | 0.246 | 0.347 | 0.326 | 0.302 | 0.260 | 0.285 | 0.417 | 0.393 | 0.317 | 0.332 | 0.395 | 0.311 | 0.394 | 0.353 | 0.343 | 0.339 | 0.356 | 0.333 | 0.316 | 0.384 |
| **BouH** | -- | -- | -- | -- | -- | -- | -- | -- | -- | 0.011 | 0.226 | 0.268 | 0.236 | 0.347 | 0.319 | 0.264 | 0.256 | 0.281 | 0.385 | 0.369 | 0.299 | 0.318 | 0.395 | 0.296 | 0.381 | 0.330 | 0.320 | 0.332 | 0.345 | 0.334 | 0.311 | 0.386 |
| **CamI** | -- | -- | -- | -- | -- | -- | -- | -- | -- | -- | 0.164 | 0.202 | 0.172 | 0.281 | 0.256 | 0.238 | 0.211 | 0.230 | 0.354 | 0.336 | 0.258 | 0.287 | 0.349 | 0.266 | 0.340 | 0.307 | 0.280 | 0.305 | 0.310 | 0.296 | 0.291 | 0.353 |
| **MasI** | -- | -- | -- | -- | -- | -- | -- | -- | -- | -- | -- | 0.074 | 0.068 | 0.103 | 0.084 | 0.127 | 0.074 | 0.086 | 0.234 | 0.163 | 0.109 | 0.179 | 0.250 | 0.150 | 0.219 | 0.216 | 0.174 | 0.199 | 0.232 | 0.224 | 0.218 | 0.300 |
| **SouL** | -- | -- | -- | -- | -- | -- | -- | -- | -- | -- | -- | -- | **0.003** | 0.115 | 0.105 | 0.152 | 0.146 | 0.143 | 0.219 | 0.203 | 0.130 | 0.180 | 0.305 | 0.171 | 0.215 | 0.212 | 0.177 | 0.213 | 0.247 | 0.284 | 0.234 | 0.329 |
| **SouH** | -- | -- | -- | -- | -- | -- | -- | -- | -- | -- | -- | -- | -- | 0.125 | 0.121 | 0.151 | 0.116 | 0.119 | 0.222 | 0.190 | 0.125 | 0.180 | 0.324 | 0.176 | 0.225 | 0.231 | 0.189 | 0.228 | 0.260 | 0.287 | 0.257 | 0.353 |
| **VicL** | -- | -- | -- | -- | -- | -- | -- | -- | -- | -- | -- | -- | -- | -- | 0.016 | 0.175 | 0.179 | 0.176 | 0.301 | 0.189 | 0.183 | 0.228 | 0.358 | 0.180 | 0.257 | 0.274 | 0.224 | 0.293 | 0.296 | 0.324 | 0.334 | 0.404 |
| **VicH** | -- | -- | -- | -- | -- | -- | -- | -- | -- | -- | -- | -- | -- | -- | -- | 0.141 | 0.147 | 0.157 | 0.282 | 0.140 | 0.134 | 0.195 | 0.314 | 0.139 | 0.222 | 0.228 | 0.175 | 0.258 | 0.253 | 0.285 | 0.279 | 0.354 |
| **SpoI** | -- | -- | -- | -- | -- | -- | -- | -- | -- | -- | -- | -- | -- | -- | -- | -- | 0.076 | 0.084 | 0.101 | 0.055 | 0.066 | 0.109 | 0.313 | 0.112 | 0.166 | 0.133 | 0.155 | 0.188 | 0.229 | 0.276 | 0.241 | 0.347 |
| **BetL** | -- | -- | -- | -- | -- | -- | -- | -- | -- | -- | -- | -- | -- | -- | -- | -- | -- | **0.004** | 0.141 | 0.086 | 0.043 | 0.131 | 0.315 | 0.143 | 0.202 | 0.181 | 0.194 | 0.185 | 0.258 | 0.274 | 0.246 | 0.347 |
| **BetH** | -- | -- | -- | -- | -- | -- | -- | -- | -- | -- | -- | -- | -- | -- | -- | -- | -- | -- | 0.141 | 0.103 | 0.053 | 0.161 | 0.328 | 0.166 | 0.209 | 0.195 | 0.203 | 0.199 | 0.286 | 0.290 | 0.272 | 0.370 |
| **AraH** | -- | -- | -- | -- | -- | -- | -- | -- | -- | -- | -- | -- | -- | -- | -- | -- | -- | -- | -- | 0.198 | 0.134 | 0.152 | 0.373 | 0.192 | 0.223 | 0.174 | 0.220 | 0.181 | 0.275 | 0.329 | 0.306 | 0.405 |
| **MouL** | -- | -- | -- | -- | -- | -- | -- | -- | -- | -- | -- | -- | -- | -- | -- | -- | -- | -- | -- | -- | 0.064 | 0.138 | 0.359 | 0.117 | 0.174 | 0.177 | 0.182 | 0.227 | 0.277 | 0.326 | 0.294 | 0.394 |
| **GudI** | -- | -- | -- | -- | -- | -- | -- | -- | -- | -- | -- | -- | -- | -- | -- | -- | -- | -- | -- | -- | -- | 0.086 | 0.281 | 0.101 | 0.131 | 0.112 | 0.123 | 0.145 | 0.208 | 0.241 | 0.184 | 0.304 |
| **HouH** | -- | -- | -- | -- | -- | -- | -- | -- | -- | -- | -- | -- | -- | -- | -- | -- | -- | -- | -- | -- | -- | -- | 0.236 | 0.058 | 0.103 | 0.049 | 0.081 | 0.108 | 0.122 | 0.226 | 0.172 | 0.265 |
| **EspI** | -- | -- | -- | -- | -- | -- | -- | -- | -- | -- | -- | -- | -- | -- | -- | -- | -- | -- | -- | -- | -- | -- | -- | 0.228 | 0.305 | 0.273 | 0.178 | 0.217 | 0.180 | 0.113 | 0.219 | 0.143 |
| **NeoB** | -- | -- | -- | -- | -- | -- | -- | -- | -- | -- | -- | -- | -- | -- | -- | -- | -- | -- | -- | -- | -- | -- | -- | -- | 0.080 | 0.060 | 0.077 | 0.106 | 0.096 | 0.200 | 0.149 | 0.231 |
| **PeyL** | -- | -- | -- | -- | -- | -- | -- | -- | -- | -- | -- | -- | -- | -- | -- | -- | -- | -- | -- | -- | -- | -- | -- | -- | -- | 0.093 | 0.067 | 0.107 | 0.107 | 0.291 | 0.211 | 0.301 |
| **TouH** | -- | -- | -- | -- | -- | -- | -- | -- | -- | -- | -- | -- | -- | -- | -- | -- | -- | -- | -- | -- | -- | -- | -- | -- | -- | -- | 0.077 | 0.112 | 0.127 | 0.239 | 0.147 | 0.267 |
| **TroH** | -- | -- | -- | -- | -- | -- | -- | -- | -- | -- | -- | -- | -- | -- | -- | -- | -- | -- | -- | -- | -- | -- | -- | -- | -- | -- | -- | 0.097 | 0.071 | 0.196 | 0.161 | 0.236 |
| **ChiL** | -- | -- | -- | -- | -- | -- | -- | -- | -- | -- | -- | -- | -- | -- | -- | -- | -- | -- | -- | -- | -- | -- | -- | -- | -- | -- | -- | -- | 0.071 | 0.207 | 0.154 | 0.189 |
| **LuzH** | -- | -- | -- | -- | -- | -- | -- | -- | -- | -- | -- | -- | -- | -- | -- | -- | -- | -- | -- | -- | -- | -- | -- | -- | -- | -- | -- | -- | -- | 0.170 | 0.153 | 0.151 |
| **TecL** | -- | -- | -- | -- | -- | -- | -- | -- | -- | -- | -- | -- | -- | -- | -- | -- | -- | -- | -- | -- | -- | -- | -- | -- | -- | -- | -- | -- | -- | -- | 0.145 | 0.145 |
| **SomI** | -- | -- | -- | -- | -- | -- | -- | -- | -- | -- | -- | -- | -- | -- | -- | -- | -- | -- | -- | -- | -- | -- | -- | -- | -- | -- | -- | -- | -- | -- | -- | 0.133 |
